# β-glucans from *Euglena gracilis* or *Saccharomyces cerevisiae* effects on immunity and inflammatory parameters in dogs

**DOI:** 10.1371/journal.pone.0304833

**Published:** 2024-05-31

**Authors:** Stephanie de Souza Theodoro, Maria Eduarda Gonçalves Tozato, Letícia Warde Luis, Camila Goloni, Lucas Bassi Scarpim, Marcelino Bortolo, Aulus Cavalieri Carciofi

**Affiliations:** 1 Veterinary Medicine and Surgery Department, School of Agricultural and Veterinary Sciences, São Paulo State University (UNESP), Jaboticabal, São Paulo, Brazil; 2 Animal Science Department, School of Agricultural and Veterinary Sciences, São Paulo State University (UNESP), Jaboticabal, São Paulo, Brazil; 3 Kemin Nutrisurance Nutrição Animal LTDA, Brasil, Vargeão, Santa Catarina, Brazil; Tokyo University of Pharmacy and Life Sciences: Tokyo Yakka Daigaku, JAPAN

## Abstract

Considering the differences in molecular structure and function, the effects of β-1,3-glucans from *Euglena gracilis* and β-1,3/1,6-glucans from *Saccharomyces cerevisiae* on immune and inflammatory activities in dogs were compared. Four diets were compared: control without β-glucans (CON), 0.15 mg/kg BW/day of β-1,3/1,6-glucans (Β-Y15), 0.15 mg/kg BW/day of β-1,3-glucans (Β-S15), and 0.30 mg/kg BW/day of β-1,3-glucans (Β-S30). Thirty-two healthy dogs (eight per diet) were organized in a block design. All animals were fed CON for a 42-day washout period and then sorted into one of four diets for 42 days. Blood and faeces were collected at the beginning and end of the food intake period and analysed for serum and faecal cytokines, *ex vivo* production of hydrogen peroxide (H_2_O_2_) and nitric oxide (NO), phagocytic activity of neutrophils and monocytes, C-reactive protein (CRP), *ex vivo* production of IgG, and faecal concentrations of IgA and calprotectin. Data were evaluated using analysis of covariance and compared using Tukey’s test (*P*<0.05). Dogs fed Β-Y15 showed higher serum IL-2 than dogs fed Β-S30 (*P*<0.05). A higher phagocytic index of monocytes was observed in dogs fed the B-S15 diet than in those fed the other diets, and a higher neutrophil phagocytic index was observed for B-S15 and B-Y15 than in dogs fed the CON diet (*P*<0.05). Monocytes from dogs fed B-S15 and B-S30 produced more NO and less H_2_O_2_ than those from the CON and B-Y15 groups (*P*<0.05). Despite in the reference value, CRP levels were higher in dogs fed B-S15 and B-S30 diets (*P*<0.05). β-1,3/1,6-glucan showed cell-mediated activation of the immune system, with increased serum IL-2 and neutrophil phagocytic index, whereas β-1,3-glucan acted on the immune system by increasing the *ex vivo* production of NO by monocytes, neutrophil phagocytic index, and serum CRP. Calprotectin and CRP levels did not support inflammation or other health issues related to β-glucan intake. In conclusion, both β-glucan sources modulated some immune and inflammatory parameters in dogs, however, different pathways have been suggested for the recognition and action of these molecules, reinforcing the necessity for further mechanistic studies, especially for *E*. *gracilis* β-1,3-glucan.

## Introduction

An increasingly adopted strategy in animal production is the use of food additives to improve the immune system, with the aim of reducing the incidence of diseases and overcoming the indiscriminate use of antibiotics and chemotherapy [1–3]. This strategy has also been explored in pet food with the aim of developing diets with health benefits in addition to an optimum nutrient supply [4–6]. Immunomodulatory compounds can beneficially modulate immune and inflammatory systems, thereby increasing host resistance [7,8]. β-glucans have been explored for this function, with interesting results for several species [9,10].

The β-glucans can be found in a variety of natural sources such as yeasts, mushrooms, bacteria, algae, barley, and oats, all of which have different structures and functions [11]. They have attracted attention because of their broad-spectrum biological activities, including anti-tumour, immunomodulatory, glycaemic, anti-aging, and anti-inflammatory properties [12–14]. However, only a few β-glucan structures, in particular β-1,3/1,6-glucans, have been studied for their immunomodulatory and inflammatory bioactivities, as they interact with membrane receptors on immune cells and induce specific biological responses [14–16].

In this context, β-1,3/1,6-glucans extracted from the cell wall of *Saccharomyces cerevisiae* have been widely investigated in veterinary medicine [17–19]. In dogs, β-1,3/1,6-glucans from *S*. *cerevisiae* stimulate interleukin (IL)-2 serum concentrations, phagocytic activity of peripheral cells, antibody production, and modulation of blood glucose concentrations [20]. In a subsequent study [21], evaluated the inclusion of two β-1,3/1,6-glucan molecules, with different purities incorporated into complete and balanced diets for dogs, piglets, mice, and chicks. Although differences in biological responses between the two molecules were observed, both modulated the immune system in a similar pattern in all tested species, inducing greater production of IL-2, a greater phagocytosis index of monocytes and neutrophils, and a greater production of antibodies, suggesting that vertebrates share similar mechanisms of β-1,3/1,6-glucan recognition and action.

In the last decade, seaweed polysaccharides have attracted interest because of their potential biological properties [22–24]. The unicellular alga *Euglena gracilis* is considered a potential source of β-1,3-glucan, particularly following the development of large-scale cultivation methods. When grown aerobically in the dark with a carbon source, high amounts of paramylon, an insoluble, non-digestible β-glucan composed of high molecular weight linear polymers of β-1,3-D-glucose, are deposited in the cytoplasm as highly crystalline discoid granules [25,26].

Studies on dried *E*. *gracilis* and purified paramylon (β-1,3-glucan) are available for some species. In mice, paramylon was tested in a model of atopic dermatitis skin lesions induced by 2,4,6-trinitrochlorobenzene, and the authors verified a reduction in the development of skin lesions, followed by inhibition of T-helper 1 and 2 cell responses [27]. Supplementation with fermented *E*. *gracilis*, containing more than 50% β-1,3-glucan was investigated through self-reported changes in upper respiratory tract infection symptoms in humans. This study provides evidence that supplementation can reduce or prevent symptoms of upper respiratory tract infection, provide immune support, and improve overall health [28].

To our knowledge, no studies on *E*. *gracilis* in dogs have been conducted. Considering the molecular differences between the β-1,3/1,6-glucans from *S*. *cerevisiae*, which present branched chains [15,29], and the high molecular weight linear polymers of β-1,3-glucans from *E*. *gracilis* [3,16], it is expected that these compounds differ in their interactions with immune cells and thus in their biological effects. Thus, we aimed to evaluate and compare the effects of β-glucans from *E*. *gracilis* and *S*. *cerevisiae* incorporated into complete and balanced diets on the immune and inflammatory activity of healthy adult dogs.

## Materials and methods

This study was conducted at the Laboratory of Research in Nutrition and Nutritional Diseases of Dogs and Cats “Prof. Dr. Flávio Prada”, School of Agricultural and Veterinary Sciences, São Paulo State University (UNESP), Jaboticabal, SP, Brazil. All animal procedures followed the ethical principles of the Brazilian College of Animal Experimentation and were approved by the Ethics Committee on the Use of Animals (Protocol no. 001974/20).

### Animals

Thirty-two adult Beagle dogs, 8 males and twenty-four females, with ages between 1 to 6 years old (2.8±2.3 years) and 10.7±1.4 kg were used. The mean body condition score was 5 on a scale of 1 to 9 [30]. Prior to the study, the dogs were subjected to physical, haematological, and serum biochemical evaluations by a veterinarian, and all were considered healthy. The animals were randomly distributed into treatments, but each group was balanced to present similar body weights and ages (*P*>0.05), and all was composed by 6 females and 2 males.

### Experimental design

The study included four experimental diets and was conducted in a randomised block design with two blocks of 16 dogs each, with four dogs per diet in each block for a total of eight animals (repetitions) per diet (treatment). The blocking factor was time because of the structure available for research. Each block lasted 84 days, with a 42-day washout period (when all animals received the control diet) and a 42-day treatment period, during which dogs were sorted into one of the four experimental diets. Immediately before (day 42) and at the end of each period of experimental diets intake (day 84) the following analyses were conducted: serum and faecal cytokines (TNF-α, IL-2, IL-6, and IL-10), faecal calprotectin and immunoglobulin A (IgA), serum C-reactive protein (CRP), phagocytic activity of peripheral monocytes and neutrophils, *in vitro* production of hydrogen peroxide and nitric oxide in cell culture by peripheral neutrophils and monocytes, and IgG production in cell culture.

The amount of food offered was initially calculated by considering the food metabolizable energy content, estimated by its chemical composition, and the individual energy requirements of laboratory dogs [31]. Food was provided once a day (at 3 p.m.), and the amount offered, and leftovers were weighed to calculate intake. The dogs were weighed weekly, and the amount of food provided was adjusted to maintain a constant body weight throughout the study. Water was provided *ad libitum*. During the study dogs were housed in kennels measuring 1.5 m × 3.5 m with a solarium and released daily in a collective playground for exercise and socialization during 4 to 6 hours a day.

### Ingredients evaluated

Two sources of β-glucan were studied. A purified source extracted from *S*. *cerevisiae* cell wall with 78.4% of β-1,3/1,6-glucans (MacroGard, Biorigin, Lençóis Paulista, Brazil), and a source constituted by dried *E*. *gracilis* with 58.5% of β-1,3-glucans (Pralisur, Kemin Industries, Iowa, USA). The β-glucan concentrations in the studied ingredients studied were analysed using an enzymatic assay (K-EBHLG, Megazyme© Ltd., Bray, Wicklow Country, Ireland) according to the manufacturer’s instructions.

### Experimental diets

A single formulation based on corn grain, poultry by-product meal, poultry fat, and sugarcane fibre was used (Table 1), balanced for adult dogs according to the nutritional recommendations of the European Pet Food Industry Federation [32].

**Table 1:** Ingredient and chemical composition of the formulation used on the study.

Ingredients	%
Corn grain	51.6
Poultry by-product meal	32.2
Poultry fat	9.2
Liquid palatant ^1^	3.0
Sugarcane fiber ^2^	2.0
Vitamin-mineral premix ^3^	0.6
Common Salt	0.5
Potassium chloride	0.5
Choline chloride	0.3
Mold inhibitor ^4^	0.1
Antioxidant ^5^	0.05
L-lysine	0.03
Analyzed chemical composition	%, as fed basis
Dry matter	92.8
Crude protein	28.8
Ash	5.7
Acid-hydrolyzed fat	17.6
Crude fiber	2.2
Calcium	1.0
Phosphorus	0.9

^1^ D’TECH 10L, Palatabilizante Líquido, SPF do Brasil Indústria e Comércio Ltda., Descalvado, Brazil.

^2^ Vit2be Fiber, Dilumix Industrial Ltda., Leme, Brazil.

^3^ Rovimix, DSM Produtos Nutricionais Brasil S.A., Jaguaré, Brazil. Added per kg of food: Vitamin A, 18,750 IU; Vitamin D3, 1,500 IU; Vitamin E, 125 IU; Vitamin K3, 1,5 mg; Vitamin B1, 5 mg; Vitamin B2, 16.25 mg; Pantothenic Acid, 37.5 mg; Vitamin B6, 7.5 mg; Vitamin B12, 45 mcg; Vitamin C, 0,125 g; Nicotinic Acid, 0.0625; Folic Acid, 0.75 mg; Biotin, 0.315 mg; Iron, 0.1 g; Copper, 9.25 mg; Manganese, 6.25 mg; Zinc, 0.15 g; Iodine, 1.875 mg; Selenium, 0.135 mg.

^4^ Mold-Zap Citrus, Alltech do Brasil Agroindustrial Ltda., Araucária, Brazil.

^5^ Banox, Alltech do Brasil Agroindustrial Ltda., Araucária, Brazil.

To obtain experimental diets, different amounts of β-glucan sources were included in the formulation, targeting to obtain specific intakes of the active compound per dog per day. The amount added was established based on a previously study [21], who suggested that 15 mg β-glucan per kg of body weight per day was an effective dosage of β-1,3/1,6-glucan for dogs. Considering the expected food intake by dogs during the study and the analysed β-glucan concentration in the products evaluated (Table 2), the following proportions was included (on an as-fed basis): control diet without inclusion of β-glucans source (CON), inclusion of 0.115% β-1,3/1,6-glucan source from *S*. *cerevisiae* cell wall of (Β-Y15), inclusion of 0.155% β-1,3-glucan source from *E*. *gracilis* (Β-S15), and inclusion of 0.310% β-1,3-glucan source from *E*. *gracilis* (Β-S30). The last treatment was designed to double the intake dosage seaweed β-glucan. The β-glucan sources addition was added replacing corn, not altering the remaining ingredients.

**Table 2:** Calculations of the amounts of β-glucans sources added in each diet (% of inclusion, as-fed basis).

Treatment name	Analysed β-glucan in the tested product	Target intake of ^1^ β-glucan (mg/kg BW/day)	β-glucan source addition in the diet
CON	-	-	-
B-Y15	78.4%	15 mg	0.115%
B-S15	58.5%	15 mg	0.155%
B-S30	58.5%	30 mg	0.31%

^1^ To achieve the target intake of β-glucan per kg of body weight (BW) per day, the mean food intake of the dogs was estimated, and the amount of the two tested sources added considering their different degree of purity.

Diets were manufactured at the Extrusion Laboratory of the School of Agricultural and Veterinary Sciences, São Paulo State University (UNESP), Jaboticabal, SP, Brazil. A single lot of raw material was used for the four experimental diets. The ingredients were weighed and mixed before being ground in a hammer mill (Tigre, Moinhos Tigre, São Paulo, SP), fitted with a 0.8 mm sieve screen size, and extruded in a single-screw extruder (Model Mex-250, Manzoni Indústria Ltda, Campinas, SP, Brazil), with an average extrusion capacity of 250 kg/h. The temperature of the extruder preconditioner was maintained at >85°C using direct steam injection. After extrusion, the kibbles were dried in a forced air dryer at 105°C for approximately 20 min and coated with poultry fat and a liquid palatant.

### Phagocytic activity

Phagocytic activity was measured at the beginning and end of each experimental period using a commercial kit (pHrodo *Escherichia coli* BioParticles; Molecular Probes Inc., Eugene, OR, USA). Blood samples were drawn via jugular venipuncture and placed in heparinised tubes. Then, 100 μL of each sample was incubated with 20 μL pHrodo *E*. *coli* BioParticles, a reagent provided by the commercial kit. For each blood sample, two sets of tubes were prepared: one was placed on ice, and the other was placed in a water bath at 37°C for 15 min. The samples were then lysed, centrifuged, and washed using reagents recommended by the manufacturer. On each collection day, two negative control samples were run: two tubes with no bioparticles, one placed on ice, and the other placed at 37°C. Samples were evaluated using a flow cytometer (FACSCanto; Becton Dickinson Immunocytometry System, Mountain View, CA, USA), and the results were reported as the percentage of fluorescence signal within the designated populations of monocytes and neutrophils. The target cell population was gated according to its volume and complexity [4,33].

### Cytokines evaluation in serum and faeces

Blood samples (3 mL) were collected via jugular venipuncture and placed in tubes without anticoagulants. Subsequently, the samples were centrifuged at 3,500 × g for 10 min (5810R; Eppendorf, Hamburg, Germany). The resulting serum was frozen and stored at -80°C until further analysis.

Fresh faeces (immediately after elimination) were collected on three consecutive days. Faecal samples were pooled by dog and period, and faecal extraction was performed using saline solution [34]. Approximately one gram of faeces was weighed and diluted in 10 mL of extraction buffer composed of 0.01 M phosphate-buffered saline (PBS) (pH 7.4), 0.5% Tween (Sigma-Aldrich, St Louis, MO, USA), and 0.05% sodium azide. After homogenisation, faecal suspensions were centrifuged at 1,500 × g for 20 min at 5°C. Then, 1 mL of the supernatant was transferred to a sterile microtube containing 20 μL of protease inhibitor cocktail (Sigma-Aldrich, St Louis, MO, USA). To remove the residues, the samples were centrifuged at 15,000 × g for 15 min at 5°C, and the supernatants were stored in microtubes at -20°C until further analysis.

The levels of interleukin (IL)-2, IL-6, IL-10, and tumour necrosis factor alpha (TNF-α) in faeces and serum were estimated using a Luminex kit specific to dogs, according to the manufacturer’s recommendations (MILLIPLEX MAP ELISA Canine Cytokine/Chemokine Magnetic Bead Panel—Immunology Multiplex Assay—Merck Millipore, St Charles, MO, USA).

### Faecal calprotectin determination

To quantify faecal calprotectin concentration, the same extracts as previously described were used, and quantification was performed using a specific ELISA kit (Cloud-Clone Corp, Houston, TX, USA). Readings were performed using an ELISA microplate reader (Awareness Technology, Florida, USA).

### Serum C-reactive protein

Serum C-reactive protein (CRP) was analysed using serum samples obtained as previously described using a specific ELISA kit (Cloud-Clone Corp, Houston, TX, USA) for dogs according to the manufacturer’s recommendations.

### Determination of *ex vivo* hydrogen peroxide (H_2_O_2_) and nitric oxide (NO) production

This analysis was previously described [4], briefly, blood samples (30 mL) were drawn via jugular venipuncture and placed in heparin-containing tubes. These samples were then mixed with 4.5 mL of Histopaque 1119 and 3 mL and Histopaque 1077 (Sigma-Aldrich, St. Louis, MO, USA) in 15-mL conical centrifuge tubes. The tubes were centrifuged at 700 × g for 30 min until two distinct opaque layers, representing mononuclear and granulocyte cells, were evident. Each layer was collected separately, transferred to a 50 mL conical centrifuge tube, and washed twice with isotonic phosphate-buffered saline by centrifugation at 360 × g for 10 min. If required, erythrocyte lysis was performed using 2 mL of ammonium-chloride-potassium solution (0.15 M ammonium chloride, 10 mM potassium bicarbonate, and 0.1 mM EDTA) for a maximum of 2 min.

The cells were suspended in complete medium (RPMI 1640; Merck KGaA, Darmstadt, Germany) supplemented with 40 mg/mL gentamicin and 10% foetal bovine serum. The cell concentration was adjusted to 3 × 10^5^ neutrophils or monocytes/ml. Subsequently, the suspensions were placed in 96-well flat plates (100 μL/well). Mononuclear cells were maintained at 37°C in a humidified 5% CO_2_ incubator (Thermo Fisher Scientific, Waltham, MA, USA) for one hour to allow monocytes to adhere to the well surface. Afterwards, the supernatants were carefully removed using a pipette and each well was replenished with complete medium.

To produce H_2_O_2_, 100 μL of the sample was added to 36 wells. Among these, 18 wells contained non-stimulated cells suspensions, while the remaining 18 wells were stimulated with LPS (10 μg/well—*E*. *coli* lipopolysaccharide, Sigma Aldrich, St. Louis, MO, USA). The plates were then incubated at 37°C in a humidified incubator with 5% CO_2_ for 36 h. H_2_O_2_ production was quantified as previously described [35,36]. Buffer solution (100 μL/well) consisting of 7.8 mL distilled water (dH_2_O), 0.8 mL of solution A (800 mL dH_2_O, 80 g NaCl, 2 g KCl, 2 g KH_2_PO_4_, 11.5 g Na_2_HPO_4_), 0.1 mL of solution B (100 mL dH_2_O, 1 g CaCl_2_), 0.1 mL of solution C (100 mL dH_2_O, 1 g MgCl_2_)_,_ 0.1 mL of phenol red (100 mL dH_2_O, 1 g phenol red), 0.1 mL of peroxidase (10 mg horseradish peroxidase, 2 mL PBS) and 1 mL of glucose (100 mL dH_2_O, 1 g glucose), was added to each well. In half of the non-stimulated wells and half of the LPS-stimulated wells, 10 μL/well phorbol myristate acetate (PMA) was added, followed by incubation at 37°C in a humidified incubator with 5% CO_2_ for 1 hour. For each dog, nine wells were used for non-stimulated cells, nine for non-stimulated cells and PMA, nine for LPS-stimulated cells, and nine for LPS-stimulated cells and PMA. After and incubation of one-hour, the reaction was stopped by the addition of 10 μL of 1 N NaOH. Absorbance was measured at 595 nm using a microplate reader (iMark Microplate Absorbance Reader 168–1135, Bio-Rad, Hercules, CA, USA). The results are reported in micromolar units of H_2_O_2_/3X10^5^ cells. A hydrogen peroxide standard curve was constructed for each plate, with a range of 0.25 to 16.00 nM of H_2_O_2_.

NO concentration was determined using a colorimetric method (GRIESS reaction) [37]. This analysis was conducted with nine repetitions for LPS-stimulated and nine for non-stimulated cells (10 μg/well *E*. *coli* lipopolysaccharide, Sigma Aldrich, St. Louis, MO, USA), resulting in 18 wells per sample. GRIESS reagent (100 μL) was added and prepared by diluting a 1:1 solution containing n-(1-naftil)-ethylenediamine diluted to 0.1% in dH_2_O and 1% sulfonamide diluted in 5% H_2_PO_4_ (both from Sigma Aldrich, St Louis, MO, USA). Absorbance was measured at 540 nm using a microplate reader (iMark Microplate Absorbance Reader 168–1135, Bio-Rad, Hercules, California, USA). The results are expressed as μM of NO/3×10^5^ cells. An NO standard curve was constructed for each plate with a range of 0.78 to 100 μM of NO.

### IgG production in cell culture

The previously described cell separation process was used for IgG analysis. Lymphocytes were cultured (3×10^5^) in the presence of LPS stimulus (5 μg/mL) for seven days and the supernatant was collected and frozen at -80°C until further analysis. The IgG concentration was analysed using a dog-specific IgG ELISA kit according to the manufacturer’s protocol (Cloud-Clone Corp, Houston, TX, USA).

### Faecal IgA

Faecal collection and extraction were performed as described previously. Faecal IgA was analysed using an ELISA kit designed for dogs (Bethyl Laboratories, Montgomery, TX, USA) following the manufacturer’s recommendations. The optical density (OD) was measured at 450 nm using a Microplate Reader (MRX TC Plus; Dynex Technology, Chantilly, VA, USA). To establish IgA concentration, OD of the samples was compared with the OD of a standard with a known concentration of IgA. The kit included a standard canine IgA sample, and seven dilutions of the standard were prepared to develop a regression curve between the OD and IgA levels [4].

### Statistical analysis

Each dog was considered an experimental unit. The sample size was established based on the results of IgA and interleukin 10 [4], considering a completely randomised block design. The test power was set at 0.8 (Opdoe package of R), α = 0.05, standard deviation = 149.2, and a standard error of approximately 31.7 for IgA. This analysis was performed using the sample size procedure of R, and a sample size of eight dogs was obtained. All the variables were tested for error normality and homoscedasticity. When necessary, logarithmic transformation (log10) was applied. Data were subjected to an analysis of covariance, considering the effects of the block and diet. Instead of examining the changes in the outcome variables, the differences between the final measurements were adjusted for baseline measures. This practice has been demonstrated to enhance the statistical power in intervention trials, as indicated in previous studies [4,38]. When the results of the F-test were significant, multiple comparisons of the means were performed using Tukey’s test (*P*<0.05). The analysis was conducted using the R software (version 4.2.0).

## Results

All dogs ingested the food properly and maintained a constant body weight throughout the study. Considering the verified intake of each experimental diet, dogs fed Β-Y15 ingested 13.7±2.3 mg of β-1,3/1,6-glucans/kg/day, Β-S15 ingested 14.4±2.9 mg of β-1,3-glucans/kg/day, and dogs fed Β-S30 ingested 27.3±5.7 mg of β-1,3-glucans/kg/day. So, the verified intake was very close to the amount planned, with similar intakes of β-glucan in the treatments B-Y15 and B-S15, and a double intake for dogs fed the treatment B-S30. The β-glucan sources were well-tolerated during the 42 days of consumption, as there were no changes in the clinical condition of the animals.

A higher monocyte phagocytic index (Table 3) was observed in dogs fed the B-S15 diet (*P*<0.05) than that in the other groups, which did not differ significantly from each other. The neutrophil phagocytic index was higher in B-Y15 and B-S15 fed dogs, and lower in dogs fed the CON diet (*P*<0.05). Among the evaluated serum cytokines (Table 4), dogs fed the Β-Y15 diet exhibited higher IL-2 concentration than animals fed the Β-S30 diet (*P*<0.05), but similar values in comparison with the CON and Β-S15 treatment. No significant differences were detected for the other cytokines evaluated in the serum (*P*>0.05). The concentration of cytokines in the faeces was similar between dogs fed different treatments (*P*>0.05; Table 5).

**Table 3:** Monocyte and neutrophils phagocytic index (% of positive cells) of dogs fed diets with the addition of different sources and amounts of β-glucans (mean and standard error of the mean).

	Diets^1^				
Item^2^	CON	Β-Y15	Β-S15	Β-S30	*p-value*
**MO**	84.5^b^±0.9	85.9^b^±0.9	88.9^a^±0.9	84.9^b^±0.9	0.019
**NE**	83.2^b^±2.1	94.8^a^±1.7	94.5^a^±1.7	90.6^a^^b^±2.0	0.001

1 CON = control, without β-glucan source addition; Β-Y15 = intake of 15mg of β-1,3/1,6-glucan/kg BW/day; Β-S15 = intake of 15mg of β-1,3-glucan/kg BW/day; Β-S30 = intake of 30mg of β-1,3-glucan/kg BW/day.

^2^ MO = monocytes; NE = neutrophils.

a, b = means in a row without a common superscript letter differ (P<0.05).

**Table 4:** Serum cytokines concentrations (pg/mL) of dogs fed diets with the addition of different sources and amounts of β-glucans (mean and standard error of the mean).

	Diets^1^				
**Item**	CON	Β-Y15	Β-S15	Β-S30	*p-value*
**IL-2**	71.9^a^^b^±25.6	172.7^a^±27.0	101.7^a^^b^±25.6	51.7^b^±23.2	0.027
**IL-6**	82.7±28.8	125.9±33.1	60.1±34.1	57.8±28.6	0.756
**IL-10**	28.5±7.4	35.1±8.0	37.5±7.4	53.0±7.3	0.136
**TNF-α** *	57.4±26.6	58.6±32.3	17.5±28.8	19.2±26.4	0.499

1 CON = control, without β-glucan source addition; Β-Y15 = intake of 15mg of β-1,3/1,6-glucan/kg BW/day; Β-S15 = intake of 15mg of β-1,3-glucan/kg BW/day; Β-S30 = intake of 30mg of β-1,3-glucan/kg BW/day.

a, b = means in a row without a common superscript letter differ (P<0.05).

* Values transformed to Log10 for statistical analysis.

**Table 5:** Cytokines (pg/g of feces, as-is basis) on the feces of dogs fed diets with the addition of different sources and amounts of β-glucans (mean and standard error of the mean).

	Diets^1^				
Item	CON	Β-Y15	Β-S15	Β-S30	*p-value*
**IL-2**	62.6±2.9	60.4±2.9	63.9±2.9	63.0±2.9	0.841
**IL-6**	17.1±1.8	17.3±1.8	20.6±1.8	17.9±1.8	0.519
**IL-10**	103.4±11.1	118.0±11.0	99.8±10.9	87.8±10.8	0.347
**TNF-α**	12.7±0.6	12.8±0.6	13.1±0.6	12.3±0.6	0.769

1 CON = control, without β-glucan source addition; Β-Y15 = intake of 15mg of β-1,3/1,6-glucan/kg BW/day; Β-S15 = intake of 15mg of β-1,3-glucan/kg BW/day; Β-S30 = intake of 30mg of β-1,3-glucan/kg BW/day.

No differences in faecal calprotectin levels were observed among treatments (*P*>0.05) (Table 6). Dogs fed both diets containing *E*. *gracilis* β-1,3-glucans presented higher CRP serum concentration than animals fed Β-Yeast (P<0.05), although all values observed was inside the reference interval for health dogs [39–41]. This may be related to the activation of the immune system, as can be observed by the higher *ex vivo* NO production by peripheral monocytes observed in dogs fed the same diet, in comparison with animals fed the CON and Β-Y15 diets (*P*<0.05; Table 7). The *ex vivo* production of NO by neutrophils did not differ significantly (*P*>0.05). For H_2_O_2_, however, the opposite was verified, with lower *ex vivo* production from monocytes of dogs fed diets containing β-1,3-glucans from *E*. *gracilis* than CON and Β-Y15 (*P*<0.05; Table 8). No differences in *ex vivo* H_2_O_2_ production by peripheral neutrophils were observed (*P*>0.05). The concentrations of IgG in the cell culture and IgA in the faeces did not differ (*P*>0.05) between dogs fed different experimental diets (Table 9).

**Table 6:** Calprotectin (μg/g of feces, as-is basis) and serum C-reactive protein (CRP; mg/L) of dogs fed diets with the addition of different sources and amounts of β-glucans (mean and standard error of the mean).

	Diets^1^				
**Item**	CON	Β-Y15	Β-S15	Β-S30	*p-value*
**Calprotectin**	0.006±0.0008	0.005±0.0007	0.006±0.0008	0.006±0.0007	0.558
**CRP**	0.80^a^^b^±0.05	0.79^a^±0.05	0.96^b^±0.05	0.97^b^±0.05	0.034

1 CON = control, without β-glucan source addition; Β-Y15 = intake of 15mg of β-1,3/1,6-glucan/kg BW/day; Β-S15 = intake of 15mg of β-1,3-glucan/kg BW/day; Β-S30 = intake of 30mg of β-1,3-glucan/kg BW/day.

a, b = means in a row without a common superscript letter differ (P<0.05).

**Table 7:** Nitrogen oxide (μM of NO/3x10^5^ cells) production in cell cultures of monocytes and neutrophils from the peripheral blood of dogs fed diets with the addition of different sources and amounts of β-glucans (mean and standard error of the mean).

	Diets^1^				
Item	CON	Β-Y15	Β-S15	Β-S30	*p-value*
**Monocytes**					
**Only cells**	9.3^b^±10.0	10.1^b^±10.0	45.9^a^±9.9	43.4^a^±9.9	0.018
**LPS** ^2^	8.7^b^±13.1	11.4^b^±13.0	56.9^a^±13.1	49.7^a^^b^±13.0	0.030
**Neutrophils**					
**Only cells**	3.1±1.2	3.0±1.2	5.8±1.3	4.5±1.3	0.463
**LPS**	3.1±1.2	2.4±1.2	4.4±1.3	4.9±1.2	0.472

1 CON = control, without β-glucan source addition; Β-Y15 = intake of 15mg of β-1,3/1,6-glucan/kg BW/day; Β-S15 = intake of 15mg of β-1,3-glucan/kg BW/day; Β-S30 = intake of 30mg of β-1,3-glucan/kg BW/day.

^2^ LPS–stimulated with lipopolysaccharide.

a, b = means in a row without a common superscript letter differ (P<0.05).

**Table 8:** Hydrogen peroxide (μM of H_2_O_2_/3x10^5^ cells) production in cell cultures of monocytes and neutrophils from the peripheral of dogs fed diets with the addition of different sources and amounts of β-glucans (mean and standard error of the mean).

	Diets^1^				
Item	CON	Β-Y15	Β-S15	Β-S30	*p-value*
**Monocytes**					
**PMA** ^2^	3.5^a^±0.3	3.3^a^^b^±0.3	1.9^b^±0.3	2.2^b^±0.3	0.002
**PMA + LPS** ^3^	3.6^a^±0.4	3.6^a^±0.4	1.6^b^±0.4	2.2^a^^b^±0.4	0.001
**Neutrophils**					
**PMA**	4.8±0.7	4.6±0.7	5.0±0.7	4.4±0.7	0.958
**PMA + LPS**	5.4±0.7	3.9±0.6	4.2±0.6	3.7±0.6	0.254

1 CON = control, without β-glucan source addition; Β-Y15 = intake of 15mg of β-1,3/1,6-glucan/kg BW/day; Β-S15 = intake of 15mg of β-1,3-glucan/kg BW/day; Β-S30 = intake of 30mg of β-1,3-glucan/kg BW/day.

^2^ PMA–stimulated with phorbol myristate acetate.

^3^ PMA + LPS–stimulated with phorbol myristate acetate and lipopolysaccharide.

a, b = means in a row without a common superscript letter differ (P<0.05).

**Table 9:** Lipopolysaccharide stimulated Immunoglobulin G concentration in cell culture (ug/mL) and immunoglobulin A (mg/g of feces dry matter) concentration on the feces of dogs fed diets with the addition of different sources and amounts of β-glucans (mean and standard error of the mean).

	Diets^1^				
Item	CON	Β-Y15	Β-S15	Β-S30	*p-value*
**IgG**	17.0±2.4	13.6±2.8	13.4±2.5	12.8±2.3	0.551
**IgA**	12.0±4.3	17.3±4.6	14.2±3.9	13.6±4.4	0.897

1 CON = control, without β-glucan source addition; Β-Y15 = intake of 15mg of β-1,3/1,6-glucan/kg BW/day; Β-S15 = intake of 15mg of β-1,3-glucan/kg BW/day; Β-S30 = intake of 30mg of β-1,3-glucan/kg BW/day.

## Discussion

The current study confirmed that β-glucan intake had an impact on some inflammatory and immune parameters in dogs, but the induced responses varied depending on the sources used. It has been reported that differences in shape, molecular weight, degree of branching, solubility, molecular structure, and extraction process determine the biological activity of β-glucan, which can significantly differ between sources and manufacturers [16,42,43].

In the present study, *S*. *cerevisiae* β-1,3/1,6-glucan intake stimulated IL-2 secretion and *ex vivo* phagocytic index of neutrophils without increasing calprotectin in faeces, serum CRP, or NO and H_2_O_2_ *ex vivo* production. The increase in IL-2 (in line with the numerical, although not statistically significant, increase in serum IL-6) signals the activation of innate immunity, as previously reported in many studies [9,20,21,44–46]. The enhancement of innate immune function through the increased phagocytic activity of neutrophils was also evident in the present study [47,48]. Increased serum IL-2 levels regulate the growth, proliferation, and differentiation of T-cells [49,50]. In addition, IL-2 may be involved in the stimulation of natural killer cells and cytotoxicity of monocytes [51,52]. In clinical practice, there have been reports of treatments for autoimmune diseases, human immunodeficiency virus infection, and chemotherapy for metastatic neoplasms with direct administration of IL-2 [52–54], although no disease has been attributed to IL-2 deficiency or excess [52]. This may suggest a possible role of *S cerevisiae* β-1,3/1,6-glucan in these conditions, open opportunities of studies.

When included in the diet, β-1,3/1,6-glucans reach the small intestine intact and are absorbed by Peyer’s patches, where they are recognised by circulating antigen-presenting cells (dendritic cells or M cells) and present to other immune cells [16,55]. β-1,3/1,6-glucans are microbial structures recognised as pathogen-associated molecules that initiate an immune response. This recognition is dependent on identification and binding to different cell membrane receptors [9,16]. The main receptor for β-1,3/1,6-glucans is dectin-1, which recognises its triple-helix conformation [56]; however, lactosyl ceramide, elimination receptors, and complement receptor 3 may also be involved [9,57,58]. These receptors are found on various immune cells including monocytes, macrophages, dendritic cells, neutrophils, and natural killer cells [16,59,60]. The interaction between β-1,3/1,6-glucans and these receptors activates multiple and complex signalling cascades that induce pathogen phagocytosis, generation of a respiratory burst, microbial death, and release of chemical mediators that activate and recruit other immune cells [61,62].

The phagocytic responses of monocytes and neutrophils are significantly influenced by β-glucan consumption in different ways. In the present study *E*. *gracilis* β-1,3-glucans treatment stimulated monocyte phagocytosis, while neutrophils phagocytosis was stimulated by both *E*. *gracilis* β-1,3-glucan and *S*. *cerevisiae* β-1,3/1,6-glucan. Phagocytosis is first line of defence of the body when faced with an invading agent, and these results demonstrate a relevant effect of both β-glucans, corroborating previous findings in different species [21,63] and also in dogs [20,21].

The authors [64] measured the cytokines IL-6, IL-8, IL-10, and TNF- α in dog faeces by an ELISA method, considering it a potential technique for a non-invasive biomarker of inflammation in dogs. However, in the current study, no significant differences were detected between the treatment groups. This lack of an effect may be explained by the different stimuli, experimental conditions, and methods used (in this case, the MULTIPLEX Kit). Only healthy dogs were included in the present study, and the β-glucans intake did not induce an inflammatory condition, as calprotectin did not change and CRP levels, although higher in dogs fed β-1,3-glucans, were inside the reference value for healthy dogs [39,40,41], and possibly the effect on faecal cytokines was too low or absent in this situation.

The intake of *E*. *gracilis* β-1,3-glucans did not affect serum cytokine levels, highlighting the importance of properly characterising the actions of different β-glucan molecules. These results are in line with the findings of another study [65], which compared 13 different β-glucan sources using human whole blood cell cultures and found no stimulation of cytokine production by *E*. *gracilis* β-1,3-glucan.

However, a significant increase in *ex vivo* NO production was observed in dogs fed diets containing *E*. *gracilis* β-1,3-glucans, which was not observed for β-1,3/1,6-glucans. This effect of *E*. *gracilis* β-1,3-glucans was also noted [66] in Mollusca, suggesting the activation of a signalling cascade that may enhance the defence system of the organism. However, the activated system may be different because there is no stimulus for cytokine production, which is typically induced by β-1,3/1,6-glucans through dectin-1 recognition [46,65,67]. Therefore, other cell membrane receptors and mechanisms of action have been suggested for β-1,3-glucan recognition, whose molecular structure is linear and unbranched [68,69]. Interaction with cells expressing different receptors than dectin-1 in the intestinal lumen and Peyer’s patches was proposed [70]. One hypothesis is that the complement receptor-3 (CRP-3), found on neutrophils and monocytes, are activated facilitating phagocytosis and cytotoxic degradation [71]. Studies, however, that proposes potential molecular mechanisms of action of *E*. *gracilis* β-1,3-glucans are necessary to better understand it physiological function.

NO is an inflammatory mediator present in almost all cells in the body, and its action can modulate all immunological parameters [72]. Under physiological conditions, NO acts as a vasodilator [73], anti-tumour, immunosuppressant, pro-apoptotic factor [72], neuromodulator, and mediator of smooth muscle relaxation in the cardiovascular system and gastrointestinal tract [74]. Maintaining stable NO levels is essential for health because both deficit and excess of NO have been reported to reduce general health [73]. During an inflammatory state, pro-inflammatory cytokines stimulate NO production in monocytes, macrophages, and neutrophils, leading to a potential increase in NO levels of up to 1,000-fold [75, 76]. Since cytokines did not increase after *E*. *gracilis* β-1,3-glucans intake, it cannot be suggested that this compound induced an inflammatory status, what was reinforced by the very minor increase observed in serum CRP. *E*. *gracilis* β-1,3-glucans are much less studied than the *S*. *cerevisiae* β-1,3/1,6-glucan, with limited available publications. Although a biological effect was characterised in the present study, its extension, and implications for animal health require further investigation.

In parallel with the increase in NO, a reduction in H_2_O_2_ *ex vivo* production was observed in dogs fed with *E*. *gracilis* β-1,3-glucans. H_2_O_2_ plays a crucial role in the immune response and cell signalling, working at very low physiological concentrations (nano-to micromolar). It modulates endothelial cell function through intricate mechanisms [77,78]. A reduction in H_2_O_2_ levels suggested a balanced immune response. An increase in NO may signal the preparation of the immune system, and a reduction in H_2_O_2_ does not expose the animal to an over-stimulated immune response or its negative effects on health [72,79].

Particularly when administered in lower doses, it is suggested that β-1,3-glucans may modulate innate immunity by exerting anti-inflammatory effects, as evidenced by reductions in IL-6 and TNF-α, alongside with increase in IL-10 [70,80]. These effects may occur without compromising the immune cells’ phagocytic capabilities, as indicated by enhanced phagocytosis rates observed in neutrophils and monocytes in the present study, and without impeding their ability to eliminate pathogens, as demonstrated here by elevated NO production.

Calprotectin, a protein constituting up to 50% of cytosolic proteins in neutrophils, is resistant to bacterial degradation in the colon [81]. It was used to assess possible undesirable body responses to β-glucan intake. Their low values, similar to those of CON and within the reference range for dogs, suggest that the products were well tolerated by dogs without inducing detectable intestinal alterations [82]. CRP is also among the group of proteins used to diagnose inflammation and is considered a sensitive test for detecting inflammation and disease in dogs in clinical practice [81,83,84]. Values of up to 10 or 20 mg/L are considered normal for healthy dogs [39–41]; thus, the results of the present study were well below the reference range, indicating no systemic inflammation. There is evidence that CRP is an important regulator of inflammatory processes and is not just a marker of inflammation and disease [85]. An increase in CRP is associated with higher NO production by monocytes [76,86,87], which may at least partially explain the concurrent increase in NO and CRP levels in dogs fed *E*. *gracilis* β-1,3-glucans in the present study. Therefore, considering both factors, preparation of the immune system without evidence of over-stimulation is suggested.

The effects of both β-glucans on adaptive immunity were assessed in the present study by *ex vivo* production of IgG by lymphocyte cell culture [88]. No significant effect was found, but the limitations of this *ex vivo* approach did not allow the exclusion of an *in vivo* effect of the β-glucan sources. Faecal IgA was also assessed because of its importance in intestinal immunity [89,90] and its possible dietary effects on faecal secretion [91]. However, no effects were observed on the tested products. Although not all studies with β-glucans found an effect on IgA secretion [92], the lack of an immune challenge and the fact that only healthy dogs were used in the present study does not allow us to exclude the effect of β-glucans on this parameter. Therefore, perhaps the main limitations of the present study that need to be considered in data interpretation include the use of only healthy Beagles, the absence of *in vivo* stimulation challenging the immune system of the animals, and all constraints related to the *ex vivo* methods adopted.

The *E*. *gracilis* β-1,3-glucan was tested in the present study in two dosages. Considering the body of information for *S*. *cerevisiae* β-1,3/1,6-glucan to dogs, the first dosage was similar to proposed by some authors to this compound [21]. Authors then selected another treatment with a double of this dosage, in order to explore it effects for the specie. No clear effect of intake amount was observed, since for monocytes phagocytosis dogs fed 30 mg/kg BW/day presented a lower effect than animals fed 15 mg β-1,3-glucan/kg BW/day, for IL-2 serum concentration the effect of the higher dosage was higher than that of the lower dosage, and for CRP, NO, and H_2_O_2_ the effects on dogs were similar between the two dosages. So, it is possible that the physiological effects of *E*. *gracilis* β-1,3-glucan may differ according to their dosage, but more studies are necessary to confirm this and to propose the better inclusion to influence dog’s health.

A limitation to consider in the present study is the lack of evaluation of the sex effect on the dog’s immune responses to the intake of the two β-glucan sources evaluated. In fact, very little is known about the effects of sex on prebiotic responses in dogs [93]. Most studies or used a variable number of male and females individuals but not explored the possible differences regarding sex on outcomes [94–97], as in the present study, or selected to only use females or males [92,98–100] not allowing the comparison between sex. Even in animal models’ studies, sex effects are little explored, and authors was able to localize only one study in rats, which described differences in prebiotic metabolization by male and female rat gut communities [101]. However, as evidenced in the present study, β-glucan have direct effects on immune system not necessarily related to alteration in the microbial community in the intestine, so any transference of these findings in rats might be premature not only due to the possible differences between species but also due to the particular mechanism of action on β-glucan, which directly interacts with the immune system. In any case, the present study adequately demonstrated the effects of β-glucan in dogs, regardless of sex, as 2 males and 6 females were used in each group, but the possible effects of sex on their organic actions in dogs remain unexplored.

## Conclusion

Both β-glucan sources, *S*. *cerevisiae* and *E*. *gracilis*, have the ability to modulate immune and inflammatory parameters in dogs. The intake of *S*. *cerevisiae* β-1,3/1,6-glucan showed cell-mediated activation of the immune system, as evidenced by the increased serum levels of IL-2 and neutrophil phagocytic index, while *E*. *gracilis* β-1,3-glucan acted on the immune system by increasing the *ex vivo* production of NO by monocytes, monocyte and neutrophil phagocytic index, and serum CRP concentration. Different pathways have been suggested for the recognition and action of these molecules, reinforcing the necessity for further mechanistic studies, especially for *E*. *gracilis* β-1,3-glucan. Calprotectin and CRP values did not support inflammation or other health issues related to β-glucan source intake in the present study.

## Supporting information

S1 Table Full data set. (XLSX)
